# Microwave synthesis and thermal properties of polyacrylate derivatives containing itaconic anhydride moieties

**DOI:** 10.1186/1752-153X-6-85

**Published:** 2012-08-08

**Authors:** Sameh M Osman, Mohamed H El-Newehy, Salem S Al-Deyab, Ayman El-Faham

**Affiliations:** 1 Petrochemical Research Chair, Department of Chemistry, College of Science, King Saud University, P.O. Box 2455, Riyadh 11451, Saudi Arabia; 2 Department of Chemistry, Faculty of Science, Tanta University, Tanta 31527, Egypt; 3 Alexandria University, Faculty of Science, Chemistry Department, P. O. Box 426, Ibrahimia, Alexandria 12321, Egypt

## Abstract

**Background:**

Microwave irradiation as an alternative heat source is now a well-known method in synthetic chemistry. Microwave heating has emerged as a powerful technique to promote a variety of chemical reactions, offering reduced pollution, low cost and offer high yields together with simplicity in processing and handling. On the other hand, copolymers containing both hydrophilic and hydrophobic segments are drawing considerable attention because of their possible use in biological systems. Various copolymer compositions can produce a very large number of different arrangements, producing materials of varying chemical and physical properties. Thus, the hydrophilicity of copolymers can be modified by changing the amount of incorporated itaconic anhydride.

**Results:**

A series of methyl methacrylate (MMA) and acrylamide (AA) copolymers containing itaconic anhydride (ITA) were synthesized by microwave irradiation employing a multimode reactor (Synthos 3000 Aton Paar, GmbH, 1400 W maximum magnetron) as well as conventional method. The thermal properties of the copolymers were evaluated by different techniques. Structure-thermal property correlation based on changing the itaconic anhydride ratio was demonstrated. Results revealed that the incorporation of itaconic anhydride into the polymeric backbone of all series affect the thermal stability of copolymers. In addition, the use of the microwave method offers high molecular weight copolymers which lead eventually to an increase in thermal stability.

**Conclusions:**

Microwave irradiation method showed advantages for the produced copolymers compared to that prepared by conventional method, where it can offer a copolymer in short time, high yield, more pure compounds and more thermally stable copolymers, rather than conventional method. Also, microwave irradiation method gives higher molecular weight due to prevention of the chain transfer. Moreover, as the itaconic anhydride content increases the thermal stability and *T*_*g*_ increase due to the decrease in the crystallinity.

## Background

Microwave-assisted organic synthesis has been recognized as one of the most interesting areas of current chemical research [1-5]. Microwave heating has emerged as a powerful technique to promote a variety of chemical reactions, offering reduced pollution, low cost and offer high yields together with simplicity in processing and handling [1,6-11]. The application of microwave irradiation to organic synthesis has been the focus of considerable attention in recent years and is becoming an increasingly popular technology [12-19]. Recently, there has been growing interest in applying microwave irradiation to polymer synthesis [20-23] as it can accelerate many syntheses providing selective activation with short start-up phase and can allow fast optimization of reactions.

Itaconic anhydride (ITA) is an unsaturated dicarbonic organic anhydride with one carbonyl group conjugated to the methylene group. It can be regarded as a substituted acrylic or methacrylic derivatives. In addition, it can be obtained from renewable resources [24,25]. Also, it can be polymerized [26,27] or copolymerized with various other monomers by free radical reactions [28-33].

As a result of increasing studies of acrylate derivatives and itaconic acid polymers or copolymers, various methods for the synthesis of these polymers and their copolymers with the monomers such as acrylonitrile, styrene and acrylic esters have been reported, in most cases radical polymerizations [34-42]. Various copolymer compositions can produce a very large number of different arrangements, producing materials of varying chemical and physical properties. Thus, the hydrophilicity of copolymers can be modified by changing the amount of incorporated itaconic anhydride [27].

The present work described copolymerization of acrylic monomers such as methyl methacrylate (MMA) and acrylamide (AA) with different ratios of itaconic anhydride (ITA) through free radical copolymerization in the presence of α,α'-azobisisobutyro nitrile (AIBN). Copolymerizations were carried out in both conventional and microwave conditions. The chemical structure of the prepared copolymers was confirmed by FTIR and size exclusion chromatography (SEC) and the thermal stability were studied using thermogravimetric analysis (TGA) and differential scanning calorimetry (DSC).

## Experimental

### Materials & Equipments

Itaconic anhydride (ITA) was purchased from Fluka. Acrylamide (AA) was purchased from LKB. Methyl methacrylate (MMA) was purchased from M & B. α,α`-Azobisisobutyronitrile (AIBN) was purchased from Aldrich and was recrystallized from absolute ethanol before use. All solvents were dried before use.

The microwave irradiation employing a multimode reactor (Synthos 3000, Aton Paar GmbH, 1400 W maximum magnetron) was used to prepare the copolymers. Fourier transform infrared spectroscopy (FTIR) Spectra was recorded on Nicolet 560 Magna spectrometer. Thermal properties of the copolymers were examined through using thermogravimetric analysis (TGA) under nitrogen, from room temperature to 800°C with heating rate of 10°C/min and differential scanning calorimetery (DSC) which was carried on TA-Q500 in which Specimens of (5–10 mg) were encapsulated in aluminum pans and were heated or cooled between -25°C and 400°C under dry nitrogen atmosphere with heating rate of 10°C /min. Number-average molecular weight (*M*_*n*_) and molecular weight distribution (*M*_*w*_*/M*_*n*_) were estimated by size exclusion chromatography (SEC) which was carried on Viscotek, HT-GPC. Polystyrene standards were employed for calibration. The analysis was performed at 35°C using high-performance liquid chromatography-grade THF as the eluent. Polystyrene standards were used to calibrate the molecular weight.

### Synthesis of Copolymers

#### General Procedure for Conventional Method

In a general procedure, in a three-neck round bottomed flask fitted with a condenser, methyl methacrylate (MMA) (5.00 g, 50.0 mmol) or acrylamide (AA) (3.55 g, 50.0 mmol) was mixed with itaconic anhydride (ITA) (1.12 g, 10.0 mmol) in 2-butanone (20 mL). The reaction mixture was thoroughly purged with nitrogen for 10 min. Copolymerization was initiated by adding AIBN (0.1 g, 0.01% w/w) and then was heated at 60°C, under nitrogen atmosphere for 24 h. The reaction was cooled down to room temperature, and then the copolymer was precipitated in diethyl ether. The precipitated copolymer was filtered, washed by excess of diethyl ether and was dried in oven under vacuum at 40°C for 24 h, Table 1.

**Table 1:** Yield % of MMA/ITA and AA/ITA copolymers using microwave irradiation

**Method**	**Copolymer Code**	**MMA/ITA ratio**	**MMA (mmol)**	**ITA (mmol)**	**Yield (%)**
Conventional	PMITA 1	10: 2	50	10	44.6
Microwave	PMITA 4	10: 1	100	10	76.9
	PMITA 5	10: 2	100	20	86.0
	PMITA 6	10: 4	100	40	63.8
**Method**	**Copolymer Code**	**AA/ITA ratio**	**AA (mmol)**	**ITA (mmol)**	**Yield (%)**
Conventional	PAITA 2	10: 4	50	20	09.3
Microwave	PAITA 7	10: 1	50	5	92.5
	PAITA 8	10: 2	50	10	69.8
	PAITA 9	10: 4	100	40	57.0

#### General Procedure for Microwave-Assisted Synthesis

Employing a multimode reactor (Synthos 3000, Aton Paar GmbH, 1400 W maximum magnetron); the initial step was conducted with 4-Teflon vessels rotor (MF 100) that allow processing four reactions under the same conditions. Each vessel has itaconic anhydride (ITA) was mixed with methyl methacrylate (MMA) or acrylamide (AA) in specific ratio (10:1, 10:2, and 10:4), in the presence of (0.1 g) AIBN and 2-butanone as a solvent (Table 1). The individual vessels were purged with nitrogen gas for 10 min and then were placed in the corresponding rotor, fixed by screwing down the upper rotor place, and finally the rotor was closed with a protective hood. After heating the vessels for 5 min. at 140°C and hold at the same temperature for 5 min (~2 bar pressure, 400 W). Cooling was accomplished by a fan (5 min). The final product was washed with diethyl ether, and was dried in oven under vacuum at 40°C for 24 h.

## Results and Discussion

**Scheme 1:**
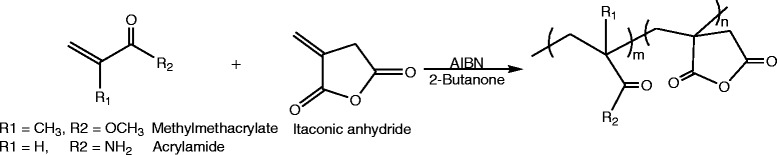
Copolymerization of itaconic anhydride with acrylic monomers.

The copolymerization reaction of methyl methacrylate and acrylamide with itaconic anhydride was carried out by two different methods: a) conventional method using of 2-butanone as a solvent and AIBN as initiator for free radical polymerization at 60°C, under nitrogen atmosphere for 24 h and b) Employing a microwave synthesis using a multimode reactor (Synthos 3000, Aton Paar GmbH, 1400 W maximum magnetron), in the presence of AIBN for 5 min. at 140°C and 400 W, Scheme 1. The prepared copolymers were characterized using FT-IR spectra, TGA, DSC, SEC, and elemental microanalysis.

### Fourier Transform Infrared Spectroscopy (FT-IR)

**Figure 1:**
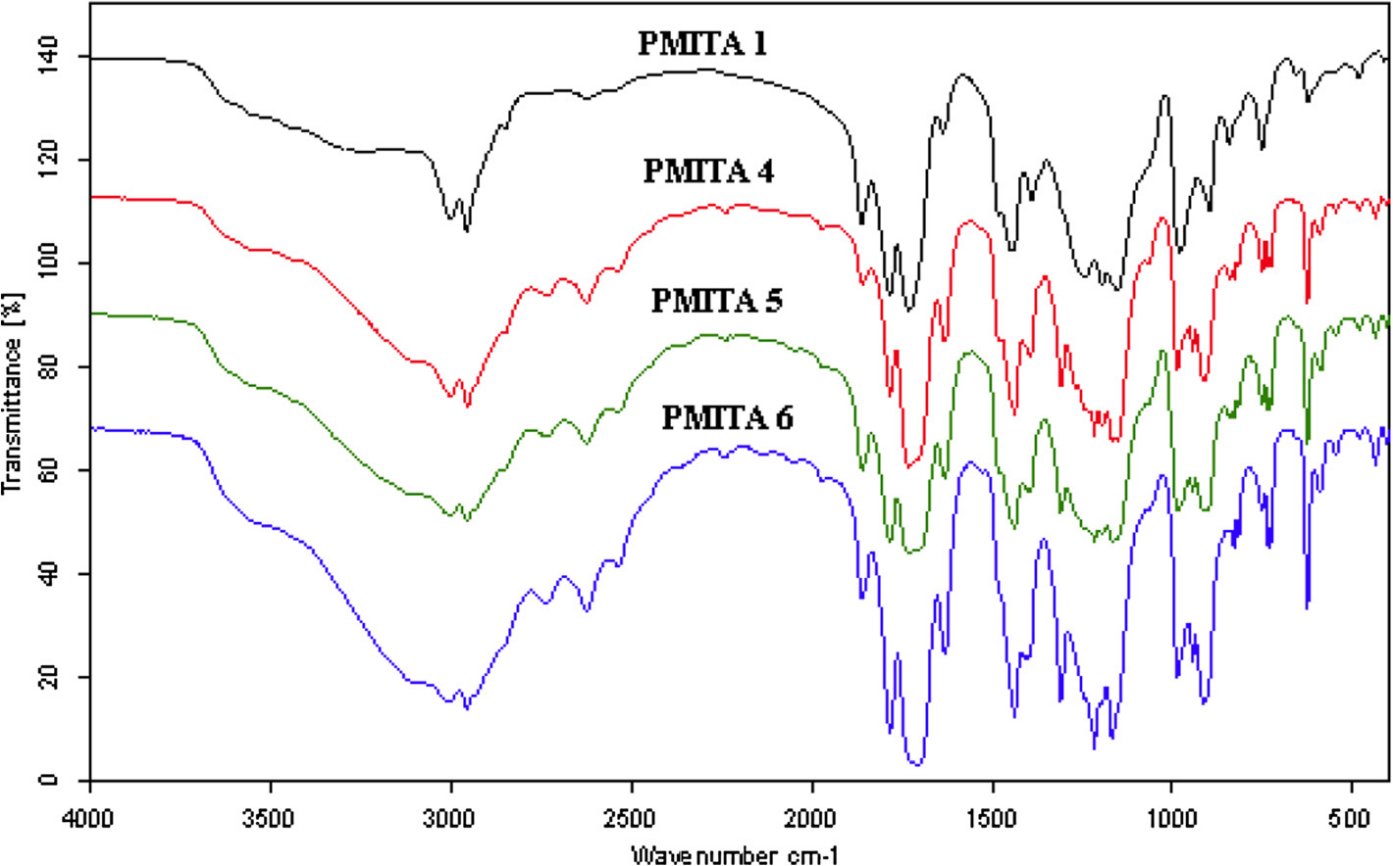
FT-IR spectra of PMITAs copolymers.

**Figure 2:**
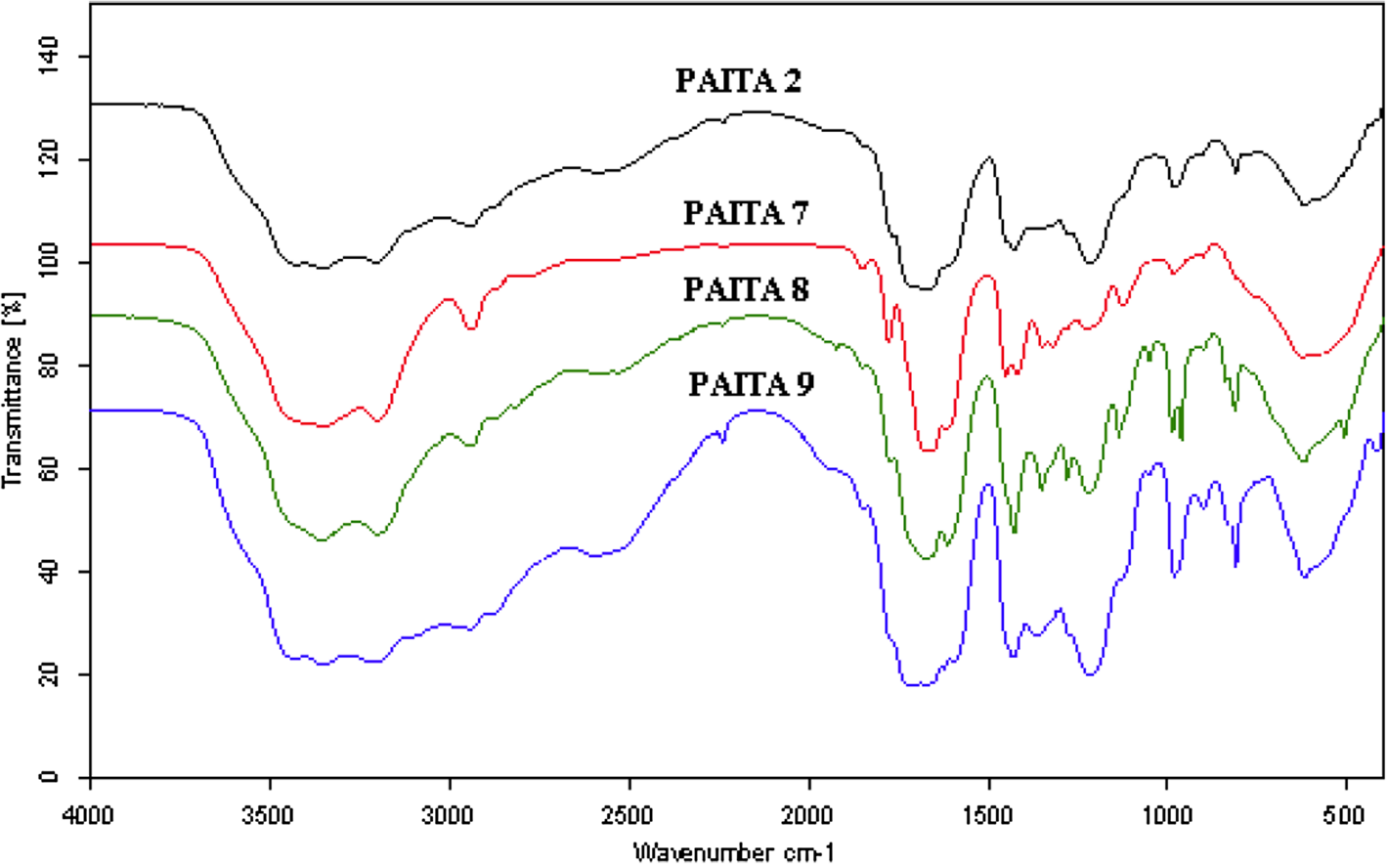
FT-IR spectra of PAITAs copolymers.

The FTIR spectra of itaconic anhydride copolymers, Figures 1 and 2 showed the absorption peaks for the ITA at 1783 and 1860 cm^-1^ (C = O symmetric and asymmetric stretching of the 5-member anhydride unit), 1662 cm^-1^ (C = C stretching), and 1400 cm^-1^ (=CH_2_ in plane deformation), and for methyl methacrylate at 1731 cm^-1^ (C = O) and for the acrylamide at 1640 cm^-1^ and at 3349 cm^-1^ originates from the secondary amines in acrylamide. The anhydride peaks indicate that the anhydride remained intact in the copolymer, and the shift to higher frequency (higher energy) of the ester and amide carbonyl stretch is consistent with the elimination of the conjugation of the vinyl and carbonyl double bonds.

The intensity of the anhydride peaks was increased as the ratio of ITA increased in the copolymer, Figures 1 and 2. Copolymers prepared by both conventional method and microwave irradiation showed the same spectral characterization.

### Thermogravimetric Analysis (TGA)

**Figure 3:**
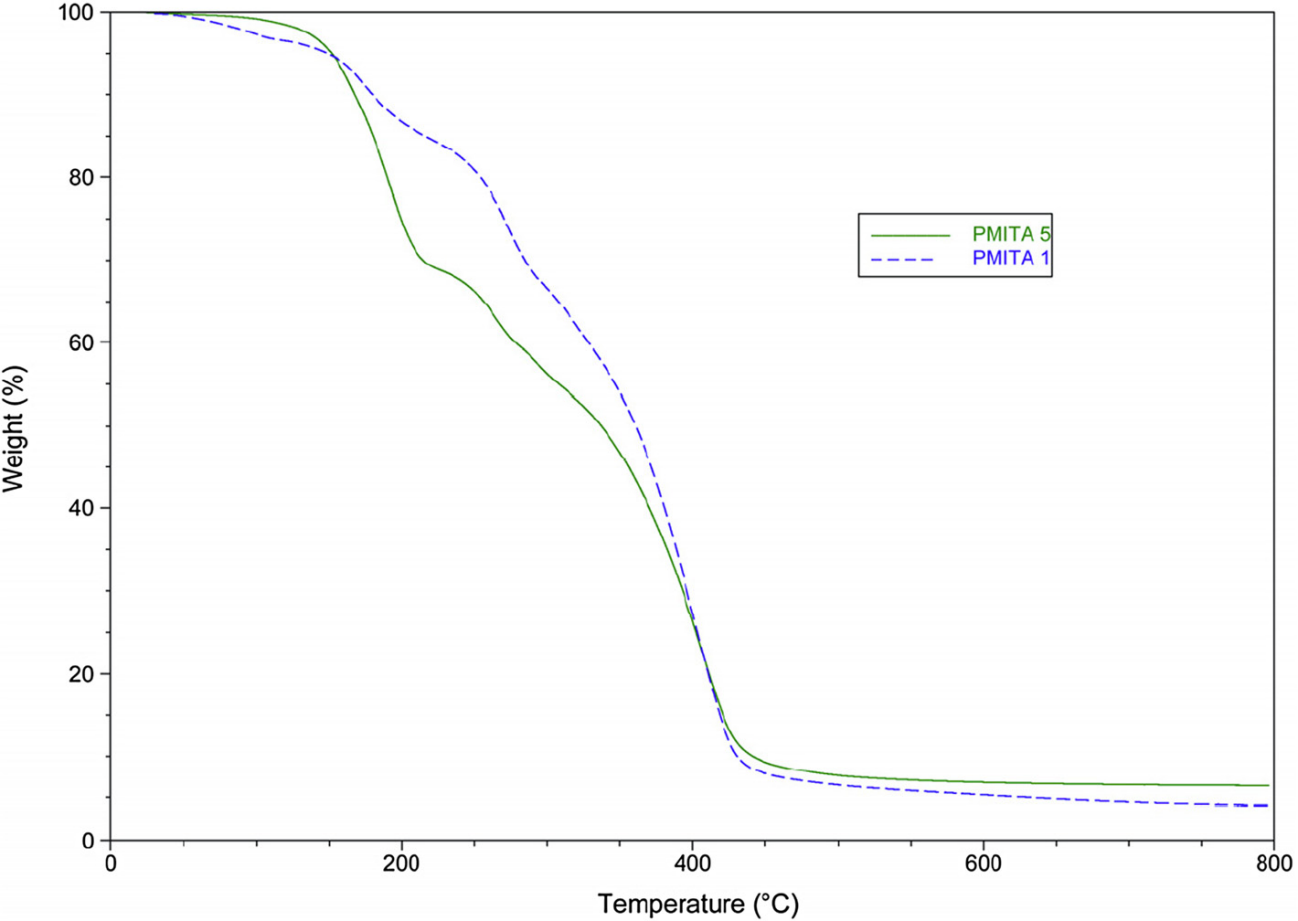
TGA of copolymers PMITA1 and PMITA5.

**Figure 4:**
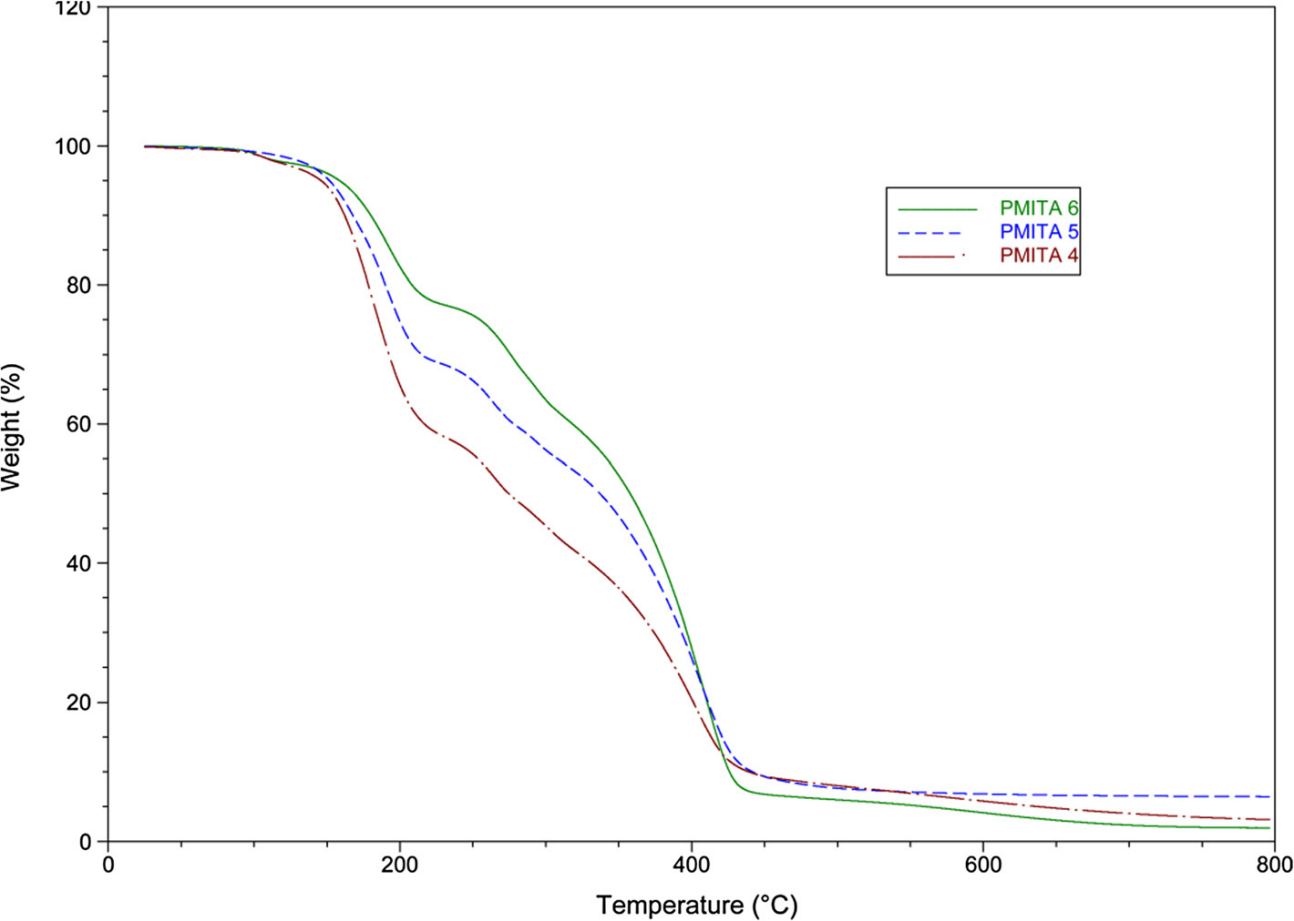
TGA thermogram of copolymers PMITA4-6.

**Figure 5:**
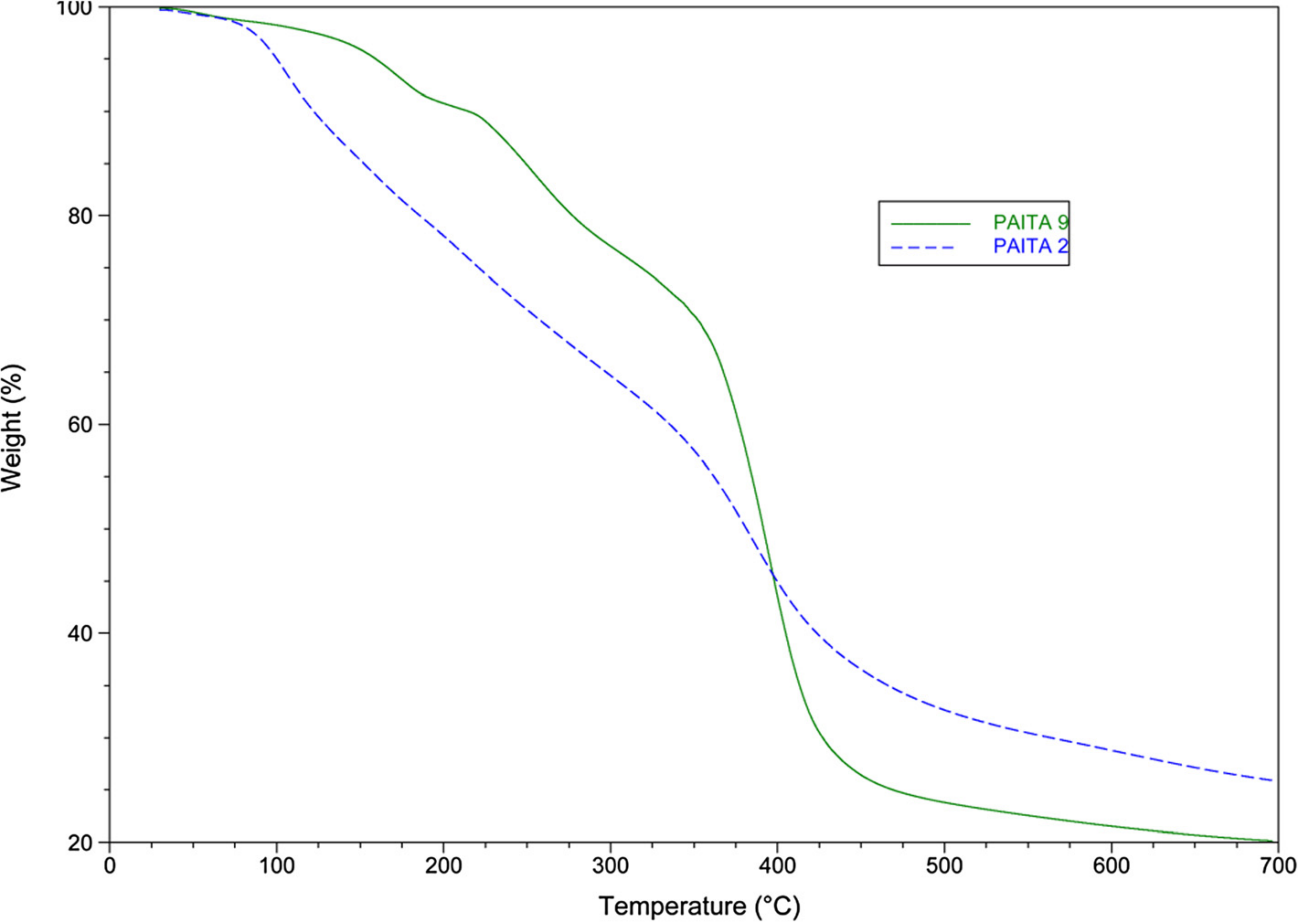
TGA thermogram of copolymers PAITA2 and PAITA9.

**Figure 6:**
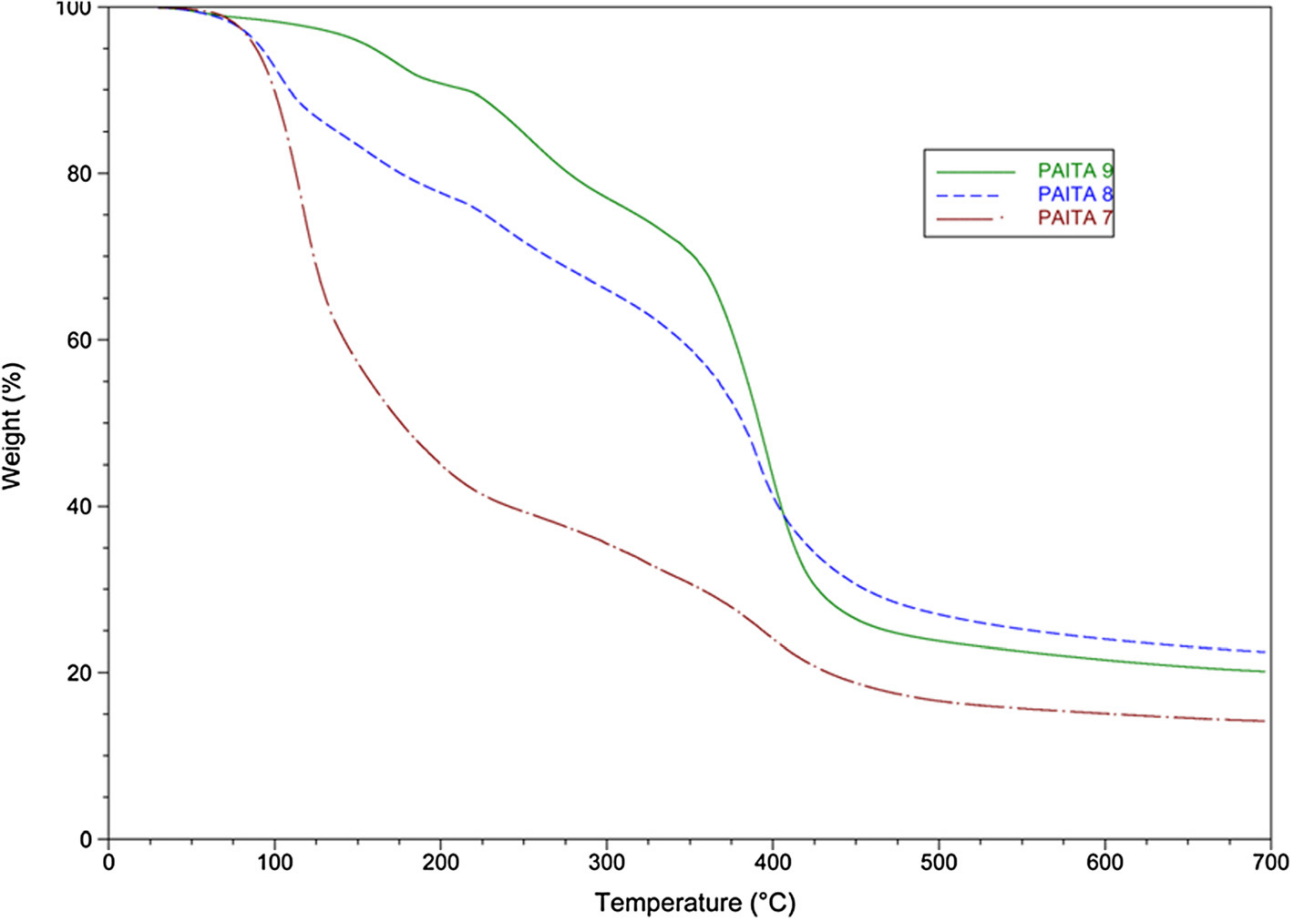
TGA thermogram of copolymers PAITA7–9.

The thermal properties of the prepared copolymers were evaluated by thermogravimetric analysis (TGA) Figures 3-6, in which the weight of a sample is measured as a function of temperature whilst it is subject to a controlled heating program. Thermal results revealed that the prepared copolymers have high thermal stabilities. Structure thermal property correlation based on changing of the itaconic anhydride ratios, as a single structural modification, demonstrated an interesting connection between itaconic anhydride ratios and thermal properties.

For copolymers PMITA1 & PMITA5, the thermogram, Figure 3, showed that the prepared copolymers were degraded in four similar degradation steps. The first step with weight loss of 2.42% for conventional method at 70-115°C and 21.27% for microwave-assisted method at 130-225°C which is attributed to water evaporation and decarboxylation which is more clear in the prepared copolymers by microwave irradiation, respectively. The last stage exhibited subsequent major degradation process between 331-445°C (87.13%, wt loss) and between 331-445°C (80.71%, wt loss) for PMITA1 and PMITA5, respectively.

For the effect of the percentage of itaconic anhydride, Figure 4, the thermogravimetric data showed that the thermal stability of copolymer PMITA6 is higher than both copolymers PMITA4 and PMITA5 due to the increase in the itaconic anhydride content as a bulk group which leads to an increase in the thermal stability.

For copolymers PAITA2 and PAITA9, the thermograms, Figure 5, showed that they were degraded via two main stage degradation processes; the first step for copolymer PAITA2 is between 100 to 110°C with weight loss of 7.53%, while the last step occurs in a range of 380 to 440°C with weight loss of 62.39%. For copolymer PAITA9, we find the first degradation step is between 115 to 120°C with weight loss of 6.19%, while the last degradation step is between 490 to 445°C with weight loss of 61.68%. The thermogram showed the major weight loss within the temperature range 331–507°C and the temperature for a maximum decomposition was 559 and 661°C for copolymers PAITA2 and PAITA9, respectively.

For the effect of the percentage of itaconic anhydride, Figure 6, the thermogravimetric data showed that the thermal stability of copolymer PAITA9 is higher than copolymer PAITA7 due to the increase in the itaconic anhydride content as a bulk group which leads to an increase in the thermal stability as described earlier.

Considerable differences in the thermal decomposition of the prepared copolymers are observed in terms of degradation temperature are shown in (Table 2).

**Table 2:** Information derived from TGA measurements of degradation of copolymers PMITA 1, 4-6 and PAITA 2, 7-9

**Copolymer Code**	***T***_***onset***_**(°C)**	***T***_***max***_**(°C)**	***T***_***50***_**(°C)**	Residue (%)*
PMITA1	154	363	360	6.5
PMITA4	165	369	358	6.0
PMITA5	158	365	337	7.6
PMITA6	158	367	275	8.0
PAITA2	86	351	381	32.7
PAITA7	94	371	176	16.6
PAITA8	84	366	382	27.0
PAITA9	145	366	391	23.8

* At 500 °C.

The copolymerization using microwave irradiation led to an increased *T*_*onset*_ of PMITA1 compared to PMITA5 which prepared by conventional method, however, *T*_*50*_ and *T*_*max*_ decreased. While on increasing the itaconic anhydride content (PMITA4-6), *T*_*onset*_ and *T*_*50*_ decreased and *T*_*max*_ are too close.

Similarly, PAITA2, 7-9, *T*_*onset*_, *T*_*50*_ and *T*_*max*_ increased from PAITA2, which prepared by conventional method, to PAITA9, which prepared by microwave irradiation. The same conclusion was observed with increasing the itaconic anhydride content (PAITA7-9).

This could be explained by that microwave irradiation gave chance for increasing the itaconic anhydride content more than conventional method. As expected from the literature, the molecular weight of the copolymers decreased with increasing ITA concentration in the feed, which may be a consequence of the allylic hydrogen in ITA that can act as a chain transfer agent in radical polymerization [43,44].

### Differential Scanning Calorimetry (DSC)

**Figure 7:**
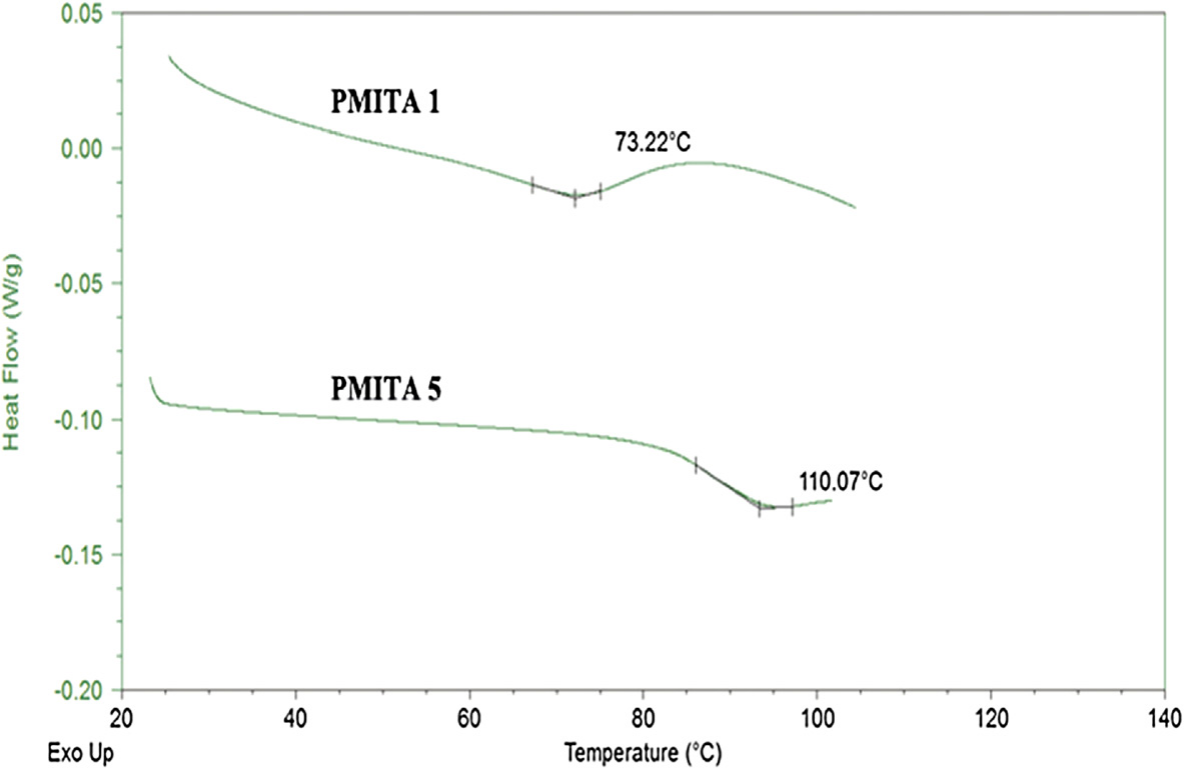
DSC thermogram of copolymers PMITA1 and PMITA5.

**Figure 8:**
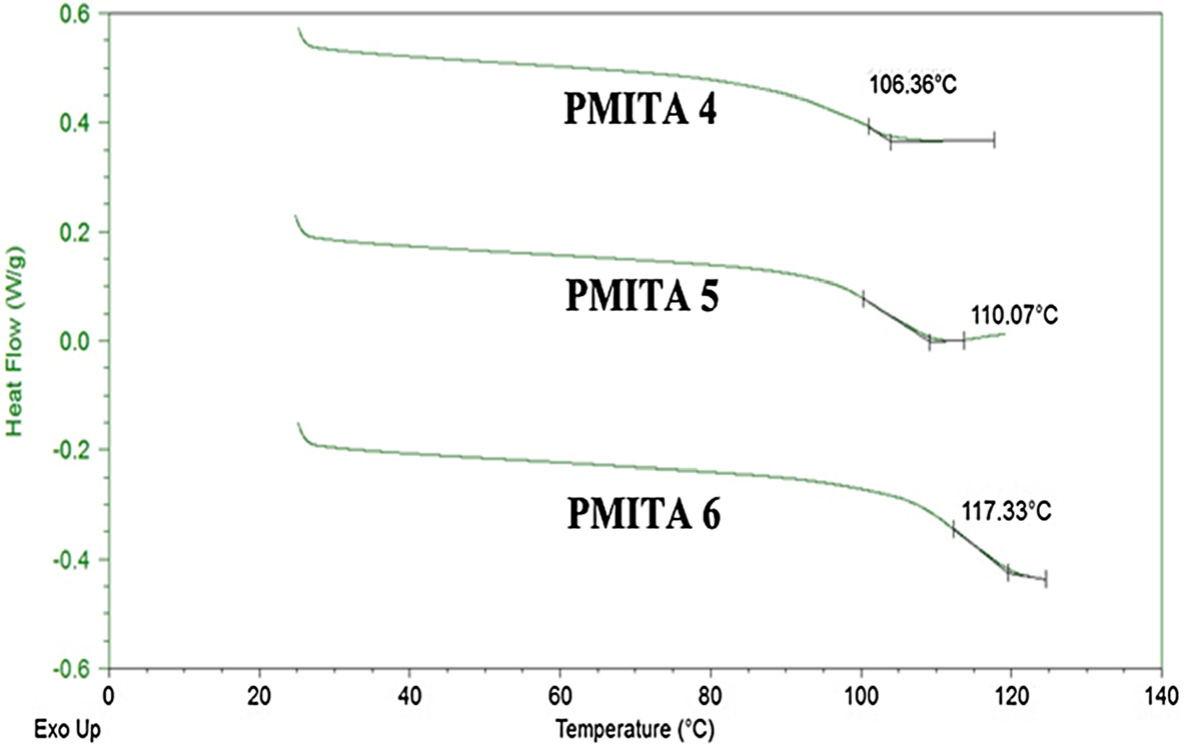
DSC thermogram of copolymers PMITA4–6.

**Figure 9:**
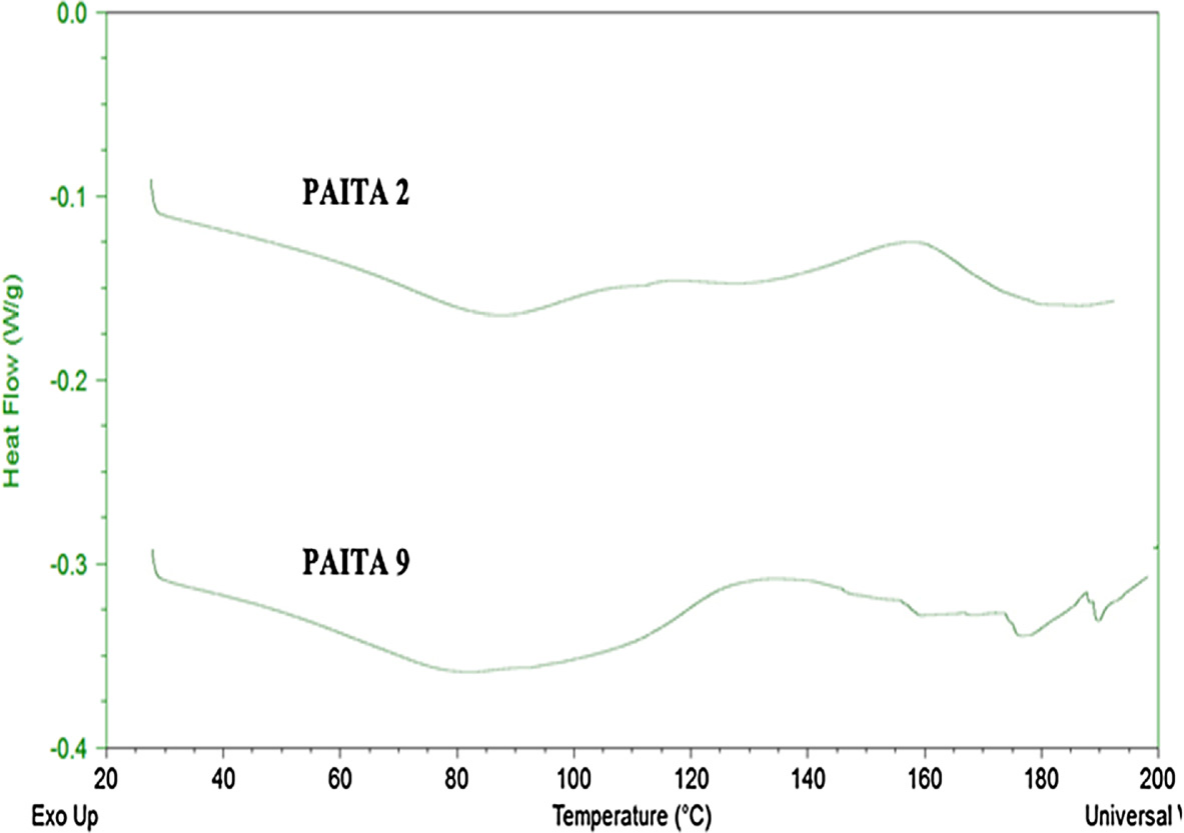
DSC thermogram of copolymers PAITA2 and PAITA9.

**Figure 10:**
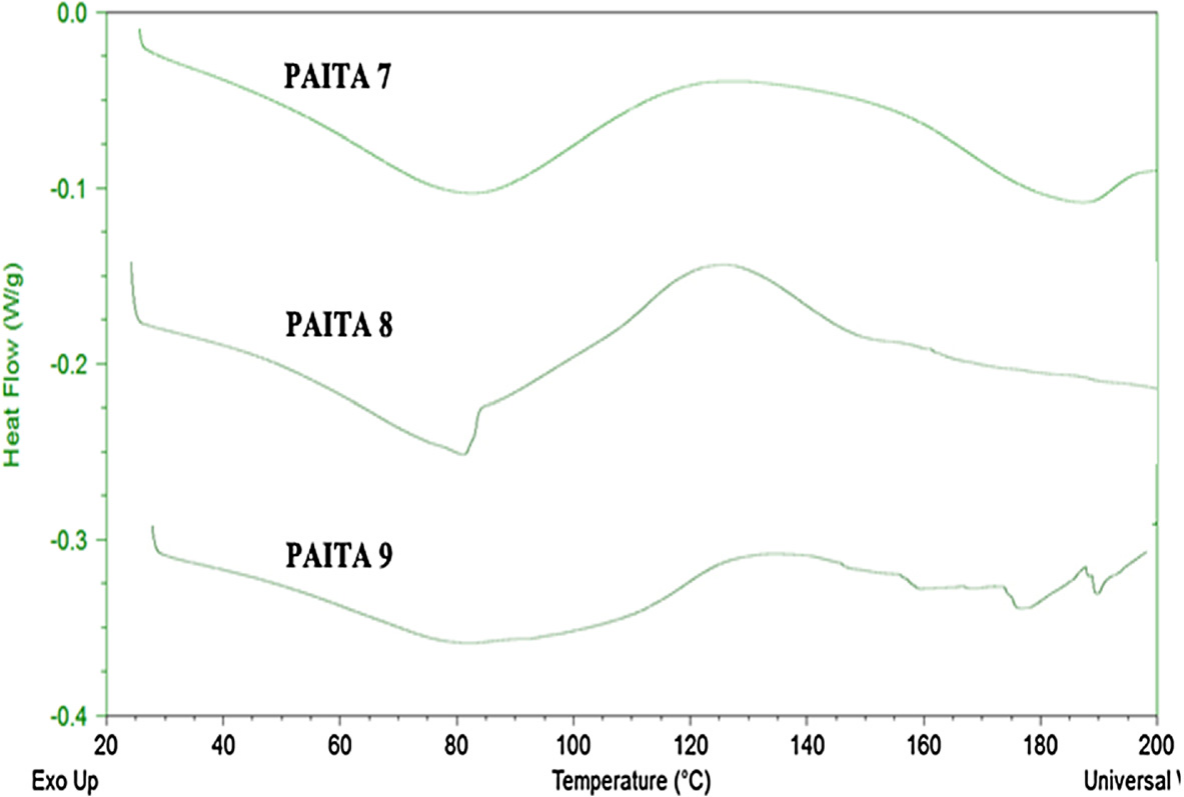
DSC thermogram of copolymers PAITA7–9.

The effect of itaconic anhydride ratio in the prepared copolymers changes on the glass transition temperature (*T*_*g*_) and the copolymer crystallinity upon thermal treatment were investigated by DSC at a heating rate of 10 °C/min, and the results are shown in Figures 7-10. The DSC traces, one transition was observed. The glass transition temperature (*T*_*g*_) was found to be 73 and 110°C, for copolymers PMITA1 and PMITA5, respectively. It was noticed that *T*_*g*_ of copolymer PMITA5 is higher than that of copolymer PMITA1, which gives another evidence for enhancing the thermal properties by using the microwave technique due to increasing in the itaconic anhydride content. On the other hand the glass transition temperature (*T*_*g*_) was found to be 106, 110, and 117°C, for copolymers PMITA4, PMITA5, and PMITA6, respectively, Figure 8. The *T*_*g*_ increased as the ITA content increase due to the decrease in crystallinity [45]. 

Figure 9 showed the DSC thermograms of copolymers PAITA2 and PAITA9 copolymers prepared by conventional and microwave method. Both show no glass transition temperature, but copolymer PAITA2 shows a hump from 30 to 100°C, this hump is assigned to the evaporation of water attached to the polar amide groups NH_2_CO- [46]. While for copolymer PAITA9 this hump starts from 30 to 120°C for the same reason. 

Moreover, for the degradation process of the copolymer PAIT2, PAIT7-9 it is clear that the effect of the hydrogen bond so during the first degradation only dehydration followed by deamination process and this stage there is no differences between the prepared polymer by convention method and microwave irradiation while at higher temperature most of the hydrogen bond were broken and the difference in the thermal stabilities and the effect of the ITA was clear [47].

For AA/ITA copolymers series PAITA7-9 prepared by microwave method, the DSC thermograms, Figure 10, showed that copolymers PAITA7-9 has no glass transition temperature, the absence of *T*_*g*_ may be due to that itaconic anhydride makes a steric hindrance for the segmental motion of the copolymer, which lead to the absence of *T*_*g*_.

**Figure 11:**
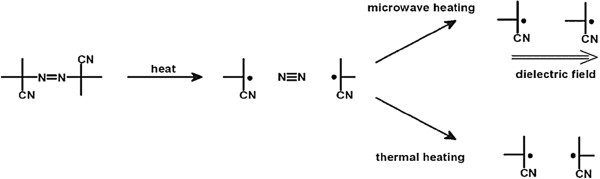
**Schematic representation of the direct orientation of radicals that are formed from the decomposition of α,α`-azoisobutyronitrile (AIBN) under microwave irradiation**[47].

Generally, the increase in the thermal stability for copolymers prepared by microwave irradiation technique compared to those prepared by conventional method is attributed to the increased radical flux under microwave irradiation as well as increasing of the itaconic anhydride content and prevention of the chain transfer in free radical polymerization. This result from the rapid orientation of the radicals that are formed from the decomposition of the α,α`-azobisisobutyronitrile as depicted in, Figure 11[43,44,48], this orientation reduces the number of direct terminations via recombination of the formed two radical fragments under microwave irradiation and thus cause a higher radical flux. The obtained high radical flux leads to the formation of high molecular weight copolymers, which result in increase in their thermal stability of the copolymers synthesized by microwave irradiation method rather than copolymers synthesized by conventional solution polymerization. 

#### The molecular weight and Elemental microanalysis Determination

**Figure 12:**
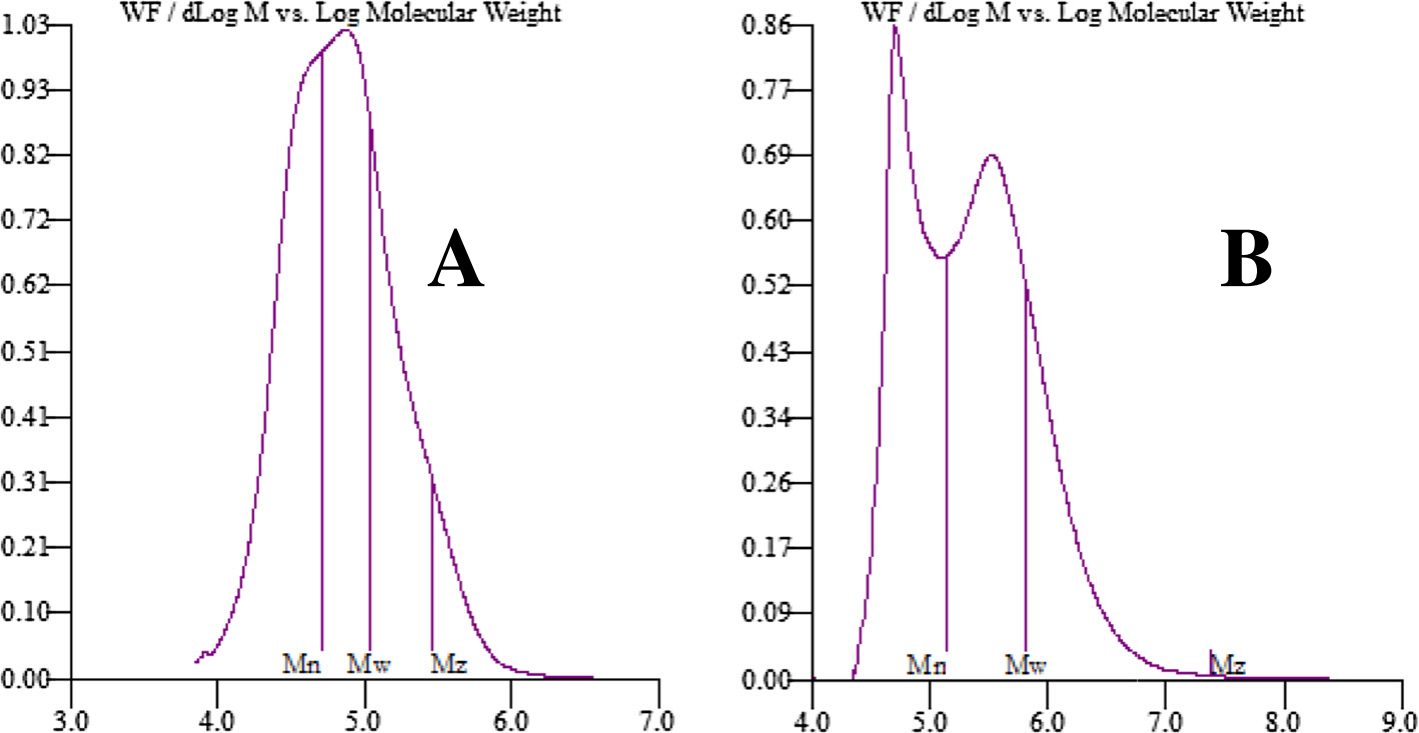
**SEC traces of copolymers a) PMITA5, b) PMITA6. For copolymer PMITA5,*M***_**n**_ **= 140,872,*M***_**w**_ **= 653.924,*M***_***w***_**/*M***_***n***_ **= 4.64. For copolymer PMITA6,*M***_**n**_ **= 50,574,*M***_**w**_ **= 288,871,*M***_**w**_**/*M***_**n**_ **= 2.14, which obtained at MMA/ITA ratio 10:2 and 10:4, respectively, in 2-butanone using microwave irradiation.**

The molecular weight of itaconic anhydride copolymers have been studied, again it is obvious that the molecular weight decrease with increasing the ratio of itaconic anhydride in the copolymer with a significant change using microwave irradiation method due to the prevention of chain transfer and increasing of the itaconic anhydride in the copolymer composition (PMITA 5), Figure 12. For SEC measurements, SEC traces showed that for copolymer PMITA5, *M*_*n*_ was 140,872 g/mol with polydispersity 4.64, while for copolymer PMITA6, *M*_*n*_ was found to be 50,574 g/mol with polydispersity of 2.14.

Moreover Figure 12B not 12A showed a bimodal population in which the first one is related to the formation of itaconic anhydride homopolymer due to the increase in itaconic anhydride ratio.

In addition, the copolymers structures and ratios were confirmed by elemental microanalysis which in a good agreement with the calculated values (Table 3).

**Table 3:** Elemental microanalysis of the prepared copolymers using microwave irradiation

**Copolymer code**	**Calculated (%)**			**Found (%)**		
	**C**	**H**	**N**	**C**	**H**	**N**
PMITA4	59.3	7.6	-	58.6	7.8	-
PMITA5	58.8	7.2	-	58.3	7.6	-
PMITA6	58.0	6.6	-	55.2	5.5	-
PAITA7	51.1	6.6	17.0	48.4	7.0	15.9
PAITA8	51.4	6.5	14.4	49.3	6.6	14.1
PAITA9	51.8	6.0	11.7	49.8	6.4	9.8

## Conclusions

Itaconic anhydride based copolymers with both methyl methacrylate and acrylamide with different ratios were successfully prepared using microwave irradiation technique. For comparison study, similar copolymers were prepared by conventional method. The obtained results showed the advantages of microwave irradiation method over conventional method. Microwave irradiation method offers the possibility of the preparation of copolymer in short time with high yield, high molecular weight and high thermal stability. Moreover, as the itaconic anhydride content increases the thermal stability and *T*_*g*_ increase due to the decrease in the crystallinity.

## Competing interests

The authors declare that they have no competing interests.

## Authors' contributions

SMO carried out all the experimental work. MHE, SSE and AEF designed the proposed methods and analyzed the data statistically together. All authors read and approved the final manuscript.

## References

[B1] Lidstrom P Tierney J Wathey B Westman J Microwave-assisted organic synthesis a review. Tetrahedron 2001 57 9225 9283

[B2] Perrux L Loupy AA Tentative realization of microwave effects in organic synthesis according to the reaction medium and mechanistic consideration Tetrahedron 2001 57 9199 9223 10.1016/S0040-4020(01)00905-X

[B3] Caddick S Microwave-assisted organic-reactions Tetrahedron 1995 51 10403 10432 10.1016/0040-4020(95)00662-R

[B4] Matloobi M Kappe CO Microwave synthesis in high-throughput environments Moving from automated sequential to microtiter plate formats 2007 2007 25 26 31

[B5] Mallakpour S Rafiee Z Application of microwave-assisted reactions in step-growth polymerization: A review Iran 2008 17 907 935

[B6] Adam D Out of the kitchen Nature 2003 421 571 572 10.1038/421571a 12571563

[B7] Loupy A Solvent-free microwave organic synthesis as an efficient procedure for green chemistry Chimie 2004 7 103 112 10.1016/j.crci.2003.10.015

[B8] Lerestif JM Toupet L Sun-bandhit S Tonnard F Bazureau JP Hamelin J A new route to 2-oxazolines, bis-oxazolines, and 2-imidazoline-5-ones from imidates using solvent-free cycloadditions: Synthesis, chemical properties, and PM3 MO calculations Tetrahedron 1997 53 6351 6364 10.1016/S0040-4020(97)00295-0

[B9] Varma RS Dahiya R Kumar S Clay catalyzed synthesis of imines and enamines under solvent-free conditions using microwave irradiation Tetrahedron Lett 1997 38 2039 2042 10.1016/S0040-4039(97)00261-X

[B10] Varma RS Clay and clay-supported reagents in organic synthesis Tetrahedron 2002 58 1235 1255 10.1016/S0040-4020(01)01216-9

[B11] Karah N Synthesis and primary cytotoxicity evaluation of new 5-nitroindole-2,3-dione derivatives Eur 2002 37 909 918 10.1016/S0223-5234(02)01416-2 12446050

[B12] Kidwai M Dry media reactions Pure Appl. Chem 2001 73 147 151 10.1351/pac200173010147

[B13] Raval JP Desai KR Synthesis and antimicrobial activity of new triazolopyridinyl phenothiazines Arkivoc 2005 xiii 21 28

[B14] Raval JP Desai KG Desai KR Neat reaction technology for the synthesis of 4-oxothiazolidines derived from 2-SHBenzothiazole and antimicrobial screening of some synthesized 4-thiazolidinones J 2006 3 233 241 10.1007/BF03247213

[B15] Raval JP Desai JT Desai CK Desai KR A comparative study of microwave assisted and conventional synthesis of 2,3-dihydro-2-aryl-4-[4-(2–oxo–2H–chromen–3–yl)–1,3-thiazol–2–ylamino]-1,5–benzothiazepines and its antimicrobial activity Arkivoc 2008 xii 233 244

[B16] Raval JP Desai KR A comparative study of microwave assisted and conventional Synthesis of novel 2-(4-diethylamino-2-hydroxyphenyl)-3-substituted-thiazolidin-4-one derivatives Chemija 2009 20 101 108

[B17] Raval JP Patel HV Patel PS Patel NH Desai KR A rapid, convenient microwave assisted and conventional synthesis of novel azetidin-2-one derivatives as potent antimicrobial agents Asian J 2009 2 171 177

[B18] Al-Hazimi HM El-Faham A Ghazzali M Al-Farhan K Microwave irradiation: A facile, scalable and convenient method for synthesis of N-phthaloylamino acids Arab 2012 5 285 289 10.1016/j.arabjc.2010.06.020

[B19] Ghazzali M El-Faham A Abd-Megeed A Al-Farhan K Microwave-assisted synthesis, structural elucidation and biological assessment of 2-(2-acetamidophenyl)-2-oxo-N-phenyl acetamide and N-(2-(2-oxo-2(phenylamino)acetyl)phenyl)-propionamide derivatives J 2012 1013 163 167

[B20] Jullien H Valot H Polyurethane curing by a pulsed microwave field Polymer 1985 26 506 510 10.1016/0032-3861(85)90149-1

[B21] Silinski B Kuzmycz C Gourdenne A Synthesis under microwaves (2.45 GHz) of polyurethane polymers-I. Model study from diisocyanate and polyethertriol prepolymers Eur. Polym. J 1987 23 273 277 10.1016/0014-3057(87)90147-9

[B22] Imai Y Nemoto H Watanabe S Kakimoto MA A new facile and rapid synthesis of aliphatic polyamides by microwave assisted polycondensation of ω-amino acids and nylon salts Polym 1996 28 256 260 10.1295/polymj.28.256

[B23] Liu Y Sun XD Xie XQ Scola DA Kinetics of the crosslinking reaction of a bisnadimide model compound in thermal and microwave cure processes J 1998 36 2653 2665 10.1002/(SICI)1099-0518(199810)36:14<2653::AID-POLA25>3.0.CO;2-G

[B24] Wallach JA Storrs CT Biodegradable polymers derived from renewable resources: highly branched copolymers of itaconic anhydride Polym. Sci 2000 University of Connecticut

[B25] Meier MAR Metzger JO Schubert US Plant oil renewable resources as green alternatives in polymer science Chem 2007 36 1788 17802 10.1039/b703294c 18213986

[B26] Papanu VD Amide-imide derivatives of homopolymers of itaconic anhydride having antitumor activity. application: EP 1983 USA: Monsanto Co 18

[B27] Milovanovic MB Trifunovic SS Katsikas L Popovic IG Preparation and modification of itaconic anhydride–methyl methacrylate copolymers J 2007 72 12 1507 1514 10.2298/JSC0712507M

[B28] Cismaru L Hamaide T Popa M Itaconic anhydride based amphiphilic copolymers: synthesis, characterization and stabilization of carboxyl functionalized PEGylated nanoparticles 2007 43 4843 4851

[B29] Shang S Huang SJ Weiss RA Synthesis and characterization of itaconic anhydride and stearyl methacrylate copolymers Polymer 2009 xxx 1 9

[B30] Erbil C Terlan B Akdemir O Gokceoren AT Monomer reactivity ratios of N-isopropylacrylamide–itaconic acid copolymers at low and high conversions Eur 2009 45 1728 1737 10.1016/j.eurpolymj.2009.02.023

[B31] Mormann W Ferbitz J Copolymers from tert-butyl methacrylate and itaconic anhydride-reactivity ratios and polymer analogous reactions Eur 2003 39 489 496 10.1016/S0014-3057(02)00246-X

[B32] Kun RC Soykan C Delibas A Study of free-radical copolymerization of itaconic acid/2-acrylamido-2-methyl-1-propanesulfonic acid and their metal chelates Eur 2006 42 625 637 10.1016/j.eurpolymj.2005.08.018

[B33] Bajaj P Paliwal DK Gupta AK Acrylonitrile-acrylic acids copolymers I synthesis and characterization J 1993 49 823 833 10.1002/app.1993.070490508

[B34] Xue W Champ S Huglin MB Observations on some copolymerisations involving N-isopropylacrylamide Polymer 2000 41 7575 7581 10.1016/S0032-3861(00)00171-3

[B35] Cowie JMG McEwen IJ Yule DJ The influence of solvent on the apparent reactivity ratios in free radical copolymerisation reactions between itaconic acid and 2-hydroxyethyl acrylate Eur 2000 36 1795 1803 10.1016/S0014-3057(99)00257-8

[B36] Uyanık N Erbil C Monomer reactivity ratios of itaconic acid and acrylamide copolymers determined by using potentiometric titration method Eur 2000 36 2651 2654 10.1016/S0014-3057(00)00045-8

[B37] Erbil C Ozdemir S Uyanık N Determination of the monomer reactivity ratios for copolymerization of itaconic acid and acrylamide by conductometric titration method Polymer 2000 41 1391 1394 10.1016/S0032-3861(99)00291-8

[B38] Virtanen J Tenhu H Studies on copolymerization of N-isopropylacrylamide and glycidyl methacrylate J 2001 39 3716 3725 10.1002/pola.10017

[B39] Devasia R Nair CPR Ninan KN Copolymerization of acrylonitrile with itaconic acid in dimethylformamide: effect of triethylamine Eur 2003 39 537 544 10.1016/S0014-3057(02)00275-6

[B40] Patel MV Dolia MB Patel JN Patel RM Synthesis and characterization of novel acrylic copolymers: determination of monomer reactivity ratios and biological activity React 2005 65 195 204 10.1016/j.reactfunctpolym.2005.08.002

[B41] Pekel N S_ahiner N Guven O Rızaev ZMO Synthesis and characterization of N-vinylimidazole–ethyl methacrylate copolymers and determination of monomer reactivity ratios Eur 2001 37 2443 2451 10.1016/S0014-3057(01)00124-0

[B42] Save NS Jassal M Agrawal AK Stimuli sensitive copolymer poly (N-tert-butylacrylamide-ran-acrylamide): synthesis and characterization J 2005 95 672 680 10.1002/app.21216

[B43] Oishi T Polymerizations and copolymerizations of N-(4-substituted phenyl)itaconimides Polym 1980 12 719 727 10.1295/polymj.12.719

[B44] Urzua M Opazo A Gargallo L Radić D Poly(N-1-alkylitaconamic acids) /poly(N-vinyl-2-pyrrolidone) blends Polym 1998 40 63 67 10.1007/s002890050224

[B45] Shang S Huang SJ Weiss RA Synthesis and characterization of itaconic anhydride and stearyl methacrylate copolymers Polymer 2009 50 3119 3127 10.1016/j.polymer.2009.05.012

[B46] Ehrenstein GW Riedel G Trawiel P Thermal analysis of plastics: Theory and practice Hanser Gardner Publications 2004 ISBN-13 9781569903629

[B47] Krušić KM Džunuzović E Trifunović S Filipović J Polyacrylamide and poly(itaconic acid) complexes Eur 2004 40 793 798 10.1016/j.eurpolymj.2003.11.016

[B48] Fellows CM Preliminary observations on the copolymerisation of acceptor monomer:donor monomer systems under microwave irradiation Central Eur J Chem 2005 3 40 52 10.2478/BF02476236

